# Antimicrobial-Resistant *Enterococcus* spp. in Wild Avifauna from Central Italy

**DOI:** 10.3390/antibiotics11070852

**Published:** 2022-06-24

**Authors:** Giulia Cagnoli, Fabrizio Bertelloni, Paolo Interrante, Renato Ceccherelli, Margherita Marzoni, Valentina Virginia Ebani

**Affiliations:** 1 Department of Veterinary Sciences, University of Pisa, 56124 Pisa, Italy; giulia.cagnoli@vet.unipi.it (G.C.); p.interrante@virgilio.it (P.I.); margherita.marzoni@unipi.it (M.M.); valentina.virginia.ebani@unipi.it (V.V.E.); 2 CRUMA-LIPU, 57121 Livorno, Italy; apusvet.cruma@libero.it; 3 Centre for Climate Change Impact, University of Pisa, 56124 Pisa, Italy

**Keywords:** *Enterococcus* spp., antimicrobial resistance, wild birds, feces

## Abstract

Bacteria of the genus *Enterococcus* are opportunistic pathogens, part of the normal intestinal microflora of animals, able to acquire and transfer antimicrobial resistance genes. The aim of this study was to evaluate the possible role of wild avifauna as a source of antimicrobial-resistant enterococci. To assess this purpose, 103 *Enterococcus* spp. strains were isolated from the feces of wild birds of different species; they were tested for antimicrobial resistance against 21 molecules, vancomycin resistance, and high-level aminoglycosides resistance (HLAR). Furthermore, genes responsible for vancomycin, tetracycline, and HLAR were searched. *E. faecium* was the most frequently detected species (60.20% of isolates), followed by *E. faecalis* (34.95% of isolates). Overall, 99.02% of the isolated enterococci were classified as multidrug-resistant, with 19.41% extensively drug-resistant, and 2.91% possible pan drug-resistant strains. Most of the isolates were susceptible to amoxicillin/clavulanic acid (77.67%) and ampicillin (75.73%), with only 5.83% of isolates showing an ampicillin MIC ≥ 64 mg/L. HLAR was detected in 35.92% of isolates, mainly associated with the genes *ant(6)-Ia* and *aac(6′)-Ie-aph(2″)-Ia*. Few strains (4.85%) were resistant to vancomycin, and the genes *vanA* and *vanB* were not detected. A percentage of 54.37% of isolates showed resistance to tetracycline; *tet(M)* was the most frequently detected gene in these strains. Wild birds may contribute to the spreading of antimicrobial-resistant enterococci, which can affect other animals and humans. Constant monitoring is essential to face up to the evolving antimicrobial resistance issue, and monitoring programs should include wild avifauna, too.

## 1. Introduction

Members of the genus *Enterococcus* are Gram-positive bacteria, common inhabitants of the gastrointestinal tract of humans and animals. Usually, they bring benefits to their host, related to probiotic activity and bacteriocins production, but occasionally, they act as pathogens causing infections in humans and animals with clinical forms ranging from mild to severe symptomatology [1].

The main issue related to enterococci is their antibiotic resistance. In fact, members of the genus *Enterococcus* are intrinsically resistant to many antimicrobials, and they have the ability to acquire and transfer genes encoding for resistance to different molecules [2].

Previous investigations have been carried out on antibiotic resistance in *Enterococcus* spp. isolated from domestic and wild mammals. Moreover, the role of poultry as a source of antibiotic-resistant enterococci has been documented, too [3,4,5,6].

The study of this bacterial population also in wild birds is pivotal, mainly to verify the rise and diffusion of resistance to new antimicrobials, which may represent serious issues in veterinary and human medicine [7].

Wild birds, in fact, usually contaminate different habitats with their droppings, and, thus, they act as a source of pathogens, including antibiotic-resistant bacteria for other animals and humans [8,9,10].

Some investigations to study enterococci in wild birds have been carried out worldwide, including in Europe. A study conducted in Illinois, U.S.A, investigated antimicrobial resistance in enterococcal strains isolated from raptors; high percentages of resistant isolates for many of the tested antimicrobials, except for penicillins and vancomycin, were found [11]. High percentages of tetracycline and erythromycin-resistant strains were recorded in enterococcal isolates from common buzzards, Passeriformes, and game birds in Portugal [12,13]. Resistance to macrolides, lincosamides, tetracyclines, and high-level aminoglycosides, as well as low resistance to β-lactams and vancomycin, were observed in *Enterococcus* spp. isolated from wild birds in Poland [14,15]. Similar results were obtained in a survey conducted in the Iberian Peninsula [16].

To the best of our knowledge, no data about the role of wild avifauna as spreaders of antimicrobial-resistant enterococci in Italy are available in the scientific literature.

To fill this gap, the aim of the present study was to investigate the possible involvement of wild Italian avifauna in the spreading of antimicrobial-resistant enterococci. For this purpose, *Enterococcus* spp. were isolated from feces collected from wild birds of different species from central Italy, and their antibiotic resistance was phenotypically and genetically evaluated.

## 2. Results

### 2.1. Enterococcus *spp.* Isolation

Enterococci were isolated from all the 103 analyzed samples. One strain from each animal was submitted to the successive analyses.

*E. faecium* was the most frequently cultured species, with 62 isolates (60.20%), followed by *E. faecalis*, with 36 isolates (34.95%). Three isolates (2.91%) were typed as *E. avium*, and two (1.94%) as *E. durans*. *E. faecium* was the most widespread species in synanthropic birds (17/18, 94.44%) and raptors (11/17, 64.71%). *E. faecalis* was more frequently isolated from aquatic birds (30/68, 44.12%) than from raptors (5/17, 29.41%) and synanthropic birds (1/18, 5.55%) (*p* < 0.05).

### 2.2. Antimicrobial Susceptibility Tests

#### 2.2.1. Agar Disk Diffusion Test

All isolates were resistant to oxacillin, whereas no antibiotics were effective against all the tested isolates. High percentages of resistance were detected for aminoglycosides. In detail, 89/103 (86.41%) isolates were resistant to neomycin, 91/103 (88.35%) to streptomycin, and 67/103 (65.05%) to gentamicin. High percentages of resistant strains were also found in testing fluoroquinolones (73/103, 70.87%, isolates resistant to enrofloxacin and 53/103, 51.46%, to ciprofloxacin), cephalothin (80/103, 77.67%, resistant strains), and clindamycin (91/103, 88.35%, resistant strains).

The most effective antibiotics were amoxicillin/clavulanic acid (80/103, 77.67%, susceptible isolates) and ampicillin (78/103, 75.73%, susceptible isolates). The results of the agar disk diffusion tests are summarized in Table 1.

#### 2.2.2. Vancomycin and Ampicillin MIC and HLAR

Among the 38 vancomycin-resistant isolates detected by the agar disk diffusion test, only 5 (5/103, 4.85%) showed a MIC higher than the cut-off (≥32 mg/L) and were classified as vancomycin resistant. In detail, one strain showed a MIC of 64 mg/L, and four strains showed a MIC greater than 256 mg/L (Table 2).

All the 24 ampicillin-resistant strains detected by the agar disk diffusion test were confirmed as ampicillin resistant, showing a MIC ≥ 16 mg/L. Among them, only six (6/103, 5.83%) isolates showed a MIC ≥ 64 mg/L: four strains of 256 mg/L and two strains > 256 mg/L (Table 2).

High-level aminoglycoside resistance was evaluated in 92, and 67 isolates were found to be resistant to streptomycin and gentamicin, respectively, by the agar disk diffusion test. Thirty-four (34/103, 33.01%) isolates showed high-level streptomycin resistance (HLSR), and fifteen (15/103, 14.56%) showed high-level gentamicin resistance (HLGR). Twelve (12/103, 11.65%) isolates showed high-level resistance to both streptomycin and gentamicin (Table 2).

#### 2.2.3. Classification of Isolates in Relation to Antimicrobial Resistance

On the basis of the antimicrobial resistance profiles obtained by the Kirby–Bauer test and MIC evaluations, isolates were classified as MDR, XDR, and possible PDR. In particular, 79/103 (76.69%) isolates were MDR, 20/103 (19.41%) XDR, and 3/103 (2.91%) possible PDR; 1 isolate could not be assigned because it was resistant only to oxacillin, neomycin, and streptomycin (Table 2).

### 2.3. Genotypic Resistance

Thirty-four strains were negative for all the investigated antibiotic resistance genes.

No strains had the genes *vanA*, *vanB*, encoding for enzymes involved in peptidoglycan modification conferring resistance to vancomycin due to modification of binding sites [17], and *tet(K)*, which encodes for an efflux pump responsible for tetracycline resistance [18].

Genes most frequently detected were *aac(6′)-li*, 55/103 (53.40%) positive isolates; *tet(M)*, 34/103 (33.01%) positive isolates; and *ant(6′)-la*, 17/103 (16.50%) positive isolates. Moreover, the genes *tet(L)*, *aac(6′)-le-aph(2″)-la*, and *tet(O),* were found in nine (8.74%), six (5.83%), and two (1.94%) isolates, respectively.

The gene *aac(6′)-li* encodes an acetyltransferase enzyme capable of modifying different aminoglycosides. The gene *ant(6′)-la* codes for a streptomycin adenyltransferase conferring high-level streptomycin resistance by the enzymatic inactivation of this molecule, while the gene *aac(6′)-le-aph(2″)-la* codes for a bifunctional modifying enzyme, possessing both acetyltransferase and phosphotransferase activities, which confer high-level resistance to gentamicin [18].

The genes *tet(M)* and *tet(O)* code for a protein that alters ribosomal conformation resulting in tetracycline resistance, while the genes *tet(L)* code for an efflux pump responsible for resistance to tetracycline, with a mechanism similar to *tet(K)* [18].

The transposon Tn916/Tn1545 was found in 12/103 (11.65%) isolates, always in association with *tet(M)* alone, except for one isolate; in this case, it was associated with *tet(L)*.

The results of the molecular analyses are summarized in Table 3.

### 2.4. Correlation between Phenotypic and Genotypic Resistance

Among the 56 tetracycline-resistant isolates, 25 (44.64%) were negative for the investigated *tet* genes: 24 (42.86%) isolates harbored 1 gene, in particular 22 had *tet(M)*, 1 *tet(L)*, and 1 *tet(O)*; 6 (10.71%) isolates had 2 genes (*tet(M)* and *tet(L)* in all 6 cases); and 1 (1.78%) isolate had *tet(M)*, *tet(L)*, and *tet(O)* genes.

All but one tetracycline-intermediate isolate was negative for the *tet* genes searched; one tetracycline-intermediate isolate harbored both *tet(M)* and *tet(L)*. Among the 27 tetracycline-susceptible isolates, 4 (14.81%) were positive for the gene *tet(M)*. Overall, *tet* genes were more frequently detected in tetracycline-resistant isolates (*p* < 0.05). The gene *Int-Tn* was detected only in tetracycline-resistant isolates.

The *tet* genes were equally distributed among tigecycline susceptible (18/36, 50.00%), intermediate (8/25, 32.00%), and resistant (10/42, 23.81%) isolates, without statistical differences (*p* > 0.05).

The gene *ant(6)-Ia*, responsible for high-level streptomycin resistance, was detected in 11/34 (33.35%) HLSR isolates and in 6/69 (8.70%) non-HLSR isolates (five streptomycin-resistant and one streptomycin-intermediate strain using the agar disk diffusion method). This gene was found more often in HLSR than in non-HLSR enterococci (*p* < 0.05). Eighteen out of the thirty-four (52.94%) HLSR positive isolates harbored the gene *aac(6′)-Ii*, five of them in association with *ant(6)-Ia*. The gene *aac(6′)-Ii* was detected in 37/69 (53.62%) non-HLSR isolates; no statistical differences were observed (*p* > 0.05).

The gene *aac(6′)-Ie-aph(2″)-Ia*, coding for high-level gentamycin resistance, was detected in 3/15 (20.00%) HLGR and in 3/88 (3.41%) non-HLGR isolates (2 gentamicin-resistant and 1 gentamicin-intermediate strains in agar disk diffusion method); a statistical difference emerged (*p* < 0.05). The gene *aac(6′)-Ii*, was detected in 6/15 (40.00%) HLGR isolates, in one case in association with *aac(6′)-Ie-aph(2″)-Ia*, and in 49/88 (55.68%) non-HLGR isolates; no statistical differences were observed (*p* > 0.05).

No associations were found between resistance to gentamicin, streptomycin, and neomycin and the presence of the gene *aac(6′)-Ii*. In particular, regarding neomycin, this gene was more often present in intermediate (11/14, 78.57%) than in resistant (44/89, 49.44%) and susceptible (0 strains) isolates (*p* < 0.05). The gene *aac(6′)-Ii* was found more often in gentamicin-susceptible (15/17, 88.24%) and intermediate (16/19, 84.21%) isolates than in gentamicin-resistant (24/67, 35.82%) isolates (*p* < 0.05). No differences emerged in relation to the distribution of the *aac(6′)-Ii* gene among streptomycin-susceptible (1/1, 100%), intermediate (6/11, 54.55%), and resistant (48/91, 52.75%) enterococci (*p* > 0.05).

## 3. Discussion

The results obtained in the current survey confirm that wild birds can act as reservoirs and spreaders of various pathogens, including antimicrobial-resistant bacteria, as reported by other authors [19,20,21,22].

Enterococci, which are usually present in the gastrointestinal tract of animals, were isolated from the feces of all examined birds. *E. faecium* was the most frequently identified species, whereas the recent literature reports *E. faecalis* as the predominant enterococcal species in wild avifauna [12,23,24,25]. However, *E. faecium* has been most frequently detected in synanthropic birds and raptors, whereas 83% of *E. faecalis* isolates have been cultured from aquatic birds. These findings show that the different prevalence of isolated enterococcal species are related not only to the geographic areas of sampling but also to the investigated avian population.

Obtained data confirm that antimicrobial resistance is largely widespread among enterococci. No antibiotics were effective against all the tested isolates, and most of the assayed antimicrobials were effective against less than 50% of isolates. Furthermore, all but one of the isolates were multidrug-resistant, with about 20% of them classified as XDR and 3% as possible PDR. Antibiotic resistance among members of the genus *Enterococcus* is due to the intrinsic resistance to several antibiotics associated with an extraordinary ability of these bacteria to acquire and share antimicrobial-resistant genes [7]. Animals, with their feces, may represent sources of enterococcal infection for humans, but they act as a “source of antimicrobial resistance genes,” too [26]. MDR and XDR enterococci are generally involved in human nosocomial infections [27]; in recent years, *Enterococcus* strains with these resistant phenotypes are frequently detected in wild animals, too, with percentages similar to our investigation [25,28,29]. Wildlife could represent the reservoir and a possible source of these bacteria, which are very hazardous to treat.

The most effective antimicrobials were ampicillin and amoxicillin/clavulanic acid. This finding is in agreement with other studies carried out worldwide on *Enterococcus* spp. isolates from wild birds [11,12,15,23]. Furthermore, in the present investigation, only 5.83% of isolates showed a MIC ≥ 64 mg/L. This value represents the breakpoint for the possibility of using ampicillin in association with aminoglycosides to treat enterococcal infection in humans [18]. Our investigation confirmed that wild birds could be carriers of ampicillin-resistant enterococci, representing a serious challenge to human therapy. Furthermore, all these strains were isolated from birds coming from the recovery center, which, in most cases, were synanthropic birds. The proximity to humans and their environments could have favored the acquisition of enterococci with these antibiotic resistances. These birds could contaminate urban/domestic settings with their feces, with the possibility of passing these strains to humans.

High percentages of enterococcal strains resistant to aminoglycosides were detected. Resistance against this class of antimicrobials in enterococci is well documented, and to date, only gentamicin and streptomycin are available for human therapy, generally in combination with a cell-wall-active molecule, such as ampicillin. This synergic effect vanishes if enterococci acquire resistance to a high level of these aminoglycosides [30,31]. In our study, high-level aminoglycoside resistance was found in 33.01% of isolates against streptomycin (HLSR) and in 14.56% of isolates against gentamicin (HLGR); 11.65% of isolates showed both HLSR and HLGR phenotypes.

Although the information available in the literature is heterogeneous, generally, other authors have described lower percentages of aminoglycoside-resistant isolates from wild birds than those reported in our study, also considering only HLAR-positive strains [11,12,14,23,32]. Similarly, investigations conducted in the same geographic area on domestic poultry and wild mammals reported lower percentages of HLAR enterococci [4,33], whereas an investigation on *Enterococcus* isolates from domestic dogs showed a high percentage of strains resistant to high-level aminoglycosides [34,35]. Considering that in the present study, HLSR were more frequently detected in isolates from synanthropic birds and raptors, the contact with humans or human environments could be a possible cause of the obtained results and a possible risk factor for the acquisition of high-resistant strains.

Acquisition of the genes *ant(6)-Ia* and *aac(6′)-Ie-aph(2″)-Ia* is the primary cause of HLSR and HLGR phenotypes, respectively [18]. Although in our investigation, these genes were more frequently detected in HLAR isolates than in non-HLAR isolates, more than 60% of high-level aminoglycoside-resistant *Enterococcus* strains did not carry them. Other authors have described strains resistant to high levels of streptomycin or gentamycin but negative for *ant(6)-Ia* and *aac(6′)-Ie-aph(2″)-Ia* genes, respectively [36,37]. Indeed, other mechanisms of resistance exist, too; these are mediated by less diffuse and investigated genes coding for aminoglycoside-modifying enzymes [30,38]. On the other hand, a small percentage of gene-positive isolates were non-HLAR. This finding could be related to the circulation of defective genes. Recently, Chen and collaborators described the presence of altered and silent *aac(6′)-Ie-aph(2″)-Ia* genes in non-HLGR *Enterococcus* isolates from a human hospital; the authors suggested that the reduced use of gentamicin in this particular setting has promoted the appearance and spreading of these defective genes among nosocomial enterococci [39]. In wild birds not directly exposed to antimicrobials, the same situation could occur.

Our data confirm that the gene *aac(6′)-li* is mainly associated with *E. faecium* [9]; this gene has been sometimes related to intrinsic aminoglycoside resistance, but our results did not show a particular association between its presence and resistance phenotype.

Decreased susceptibility to the tetracyclines class was detected. In particular, 54.37% of isolates were resistant to tetracycline. Other surveys on enterococci isolated from wild birds found percentages of resistance ranging between 30% and 90% [11,12,13,15,23]. Although the use of tetracyclines has drastically reduced in recent years, in the past, these antibiotics were largely employed in veterinary medicine, especially for the treatment of food-producing animal species [40]. The detected high resistance to the tetracyclines, as well as other antibiotics, could be related to the presence of antibiotic residues used in livestock and antimicrobial-resistant bacteria in the environment [41]. 

In the present study, more than half of tetracycline-resistant *Enterococcus* isolates had one or more *tet* genes. The most detected gene was *tet(M)*: 33.01% of all isolates and 51.86% of tetracycline-resistant enterococci. These findings confirm *tet(M)* as the prevalent gene responsible for tetracycline resistance in *Enterococcus* spp. isolated from wild birds [12,15,23] as well as from other animals [38].

Twelve (11.65%) *tet(M)* isolates were positive for the *Int-Tn* gene, too. This gene is part of the conjugative transposon Tn916/Tn1545 involved in the spreading of tetracycline resistance genes [31]. Similar prevalence of Tn916/Tn1545 have been previously found in wild birds [13,15].

In the present investigation, 34.95% of isolates sensitive to tigecycline were found, even though usually higher percentages of susceptible strains have been detected both in humans and domestic animals [42,43]. The *tet* genes were found to be equally distributed among susceptible, intermediate, and resistant isolates, confirming that these genes are not involved in tigecycline resistance [31].

The obtained data confirmed that wild birds are rarely carriers of vancomycin-resistant enterococci; only 4.85% of isolates showed resistance to this antimicrobial, in line with previous reports [11,12,13,23]. In the present investigation, only the presence of *vanA* and *vanB* was explored; these are the genes more frequently involved in acquired vancomycin resistance among enterococci [38]. None of the tested isolates had these genes; however, other resistance genes exist that could be involved [17].

A very high percentage of *Enterococcus* isolates was resistant to fluoroquinolones (70.87% to enrofloxacin and 51.46% to ciprofloxacin). Decreased susceptibility to these antimicrobials in enterococci from wild birds has been described by other authors [11,12,13,15,23]. Fluoroquinolones and other quinolones are frequently used in livestock, as well as in companion animals [40,44], and they could be frequently detected in the environment, in particular in wastewater and surface water [41].

## 4. Materials and Methods

### 4.1. Sampling

Fecal samples were collected from 103 dead wild birds belonging to different species between April 2019 and February 2020. In detail, 61 samples were from birds from a recovery center located in Tuscany, where they died of natural causes; carcasses were sent to the Laboratory of Avian Pathology (LAP) of the Department of Veterinary Sciences, University of Pisa, where they were submitted to necropsy. Rectal intestine was collected from each bird and kept at 4 °C until bacteriological analysis. For each examined bird, the species was annotated (Table 4). 

The remaining 42 fecal samples were from birds hunted during regular hunting seasons; gastrointestinal tracts were collected during evisceration by hunters and sent, under refrigeration conditions, to the LAP. Hunters provided a list with a number code of samples and the corresponding avian species (Table 4).

No animals were specifically sacrificed for this work, and, for this, no ethical approval was required.

### 4.2. Enterococcus *spp.* Isolation

All samples were maintained in refrigerated conditions and analyzed within 4 h from collection. A sterile swab was put into each fecal sample and streaked onto Kanamycin Aesculin Azide Agar (KAAA) (Oxoid Ltd., Basingstoke, UK); plates were incubated at 42 °C for 24 h. From each sample, a single isolated colony was subcultured on Mueller Hinton (MH) Agar (Oxoid Ltd.) to obtain pure cultures; only one strain from each animal sample was further processed. Obtained isolates were initially screened with Gram staining and catalase test; subsequently, the isolates were subjected to species identification using API20Strep^®^ (Biomerieux, Marcy l’Etoile, France) following the manufacturer’s protocol [45]. Typed strains were stored at −80 °C in Brain Heart Infusion (BHI) broth (Oxoid Ltd.) added with 30% glycerol.

### 4.3. Antimicrobial Susceptibility Tests

#### 4.3.1. Agar Disk Diffusion Test

One hundred and three isolates were initially evaluated for antimicrobial resistance using the Kirby–Bauer disk diffusion test, following CLSI guidelines [46]. The following 21 antimicrobials disks (Oxoid Ltd.) were employed in the assay: amoxicillin–clavulanic acid (20/10 µg), ampicillin (10 µg), cephalothin (30 µg), chloramphenicol (30 µg), ciprofloxacin (5 µg), clindamycin (2 µg), enrofloxacin (5 µg), erythromycin (10 µg), gentamicin (10 µg), linezolid (30 µg), neomycin (10 µg), nitrofurantoin (300 µg), oxacillin (1 µg), quinupristin–dalfopristin (15 µg), rifampicin (30 µg), streptomycin (10 µg), teicoplanin (30 µg), tetracycline (30 µg), tigecycline (15 µg), trimethoprim (5 µg), and vancomycin (30 µg). Obtained results were read according to CLSI and EUCAST interpretative criteria [47,48]. 

#### 4.3.2. High-Level Aminoglycoside Resistance (HLAR) and Minimum Inhibitory Concentration (MIC) for Vancomycin and Ampicillin

Isolates found to be resistant to streptomycin and/or gentamicin were tested for high-level aminoglycoside resistance (HLAR); the broth microdilution method suggested for this specific purpose by CLSI was adopted, and concentrations of 1000 µg/mL and 500 µg/mL were used as the cut-off for streptomycin and gentamicin, respectively [47].

To confirm the results obtained with the agar disk diffusion test, isolates found to be resistant to vancomycin were submitted to the MIC assay; breakpoint value was ≥32 mg/L [47,49]. To detect the exact minimum inhibitory concentration for ampicillin, isolates found to be resistant to ampicillin with the agar disk diffusion test were submitted to the MIC assay. Breakpoint was ≥16 mg/L for ampicillin, but an ampicillin MIC ≥ 64 mg/L vanishes the possibility of treatment in association with aminoglycosides in humans [47].

#### 4.3.3. Classification of Isolates in Relation to Antimicrobial Resistance

Based on phenotypic resistance results, the investigated strains were classified as multidrug-resistant (MDR), extensively drug-resistant (XDR), or pan drug-resistant (PDR), as previously proposed by Magiorakos et al. [50].

### 4.4. Genotypic Resistance

DNA extraction was carried out from each *Enterococcus* spp. isolate using a commercial kit, DNA Plus Kits (Zymo Research, Irvine, CA, USA), following the manufacturer’s guidelines.

Extracted DNA samples were submitted to different polymerase chain reaction (PCR) assays to verify the presence of the following resistance genes: *vanA*, *vanB*, *aac(6′)-Ie-aph(2″)-Ia*, *ant(6)-Ia*, *aac(6′)-Ii*, *tet(M)*, *tet(L)*, *tet(O)*, *tet(K)*, and *Int-tn*.

PCR protocols and primers previously published were adopted [51,52,53,54,55], and details are reported in the Supplementary Materials (Table S1).

### 4.5. Statistical Analyses

The obtained results were analyzed using Chi-square (X^2^) test and Fisher (F) test. Statistical tests were used to compare the distribution of enterococcal species, percentage of resistant isolates, and percentage of enterococci positive to resistant genes in relation to the provenience of birds (hunting activity or recovery center) and avian category (synanthropic birds, raptors, and aquatic birds). Furthermore, the correlations among the percentage of resistant isolates as well as the positivity to resistant genes and enterococcal species were also evaluated. Finally, the association between phenotypic and genotypic resistance was explored. The statistical significance threshold was set at a *p*-value of 0.05.

## 5. Conclusions

Enterococci are very able to acquire antimicrobial resistance, and for this, they should be included in monitoring programs as indicator bacteria.

Wild birds may be a relevant source of enterococci for other animals and humans. Some avian species, mainly synanthropic birds, live in environments shared by livestock, pets, and/or humans. On the other hand, migratory birds, mainly waterfowl, contribute to the diffusion for long distances of antimicrobial-resistant bacteria throughout migratory routes. Furthermore, hunted waterfowl could pass antimicrobial-resistant bacteria directly to people during carcass manipulation.

In the present study, high and diffuse resistances were found, especially for aminoglycosides and tetracyclines. Differences were observed comparing our results to those obtained in other studies carried out on wild avian populations from different geographic areas and studies on mammals from the same geographic area.

These differences highlighted that antimicrobial resistance is an evolving issue and constant monitoring of animals, including wildlife, is thus pivotal from a One Health perspective. From this point of view, wild birds, having varying lifestyle and dietary habits, represent excellent environmental bioindicators for the surveillance of zoonotic and non-zoonotic diseases, as well as antimicrobial resistance.

## Figures and Tables

**Table 1:** Results obtained testing 103 *Enterococcus* spp. isolates versus 21 antibiotics with the agar disk diffusion test.

Antibiotics		Susceptible		Intermediate		Resistant	
Class	Molecules	Number of Isolates	%	Number of Isolates	%	Number of Isolates	%
Ansamycins	RD	33	32.04	13	12.62	57	55.34
Phenicols	C	58	56.31	16	15.53	29	28.16
Oxazolidinones	LZD	43	41.75	17	16.50	43	41.75
Nitrofurantoins	F	43	41.75	19	18.45	41	39.81
Folate pathway antagonists	W	2	1.94	57	55.34	44	42.72
Aminoglycoside	N	0	0.00	14	13.59	89	86.41
	CN	17	16.50	19	18.45	67	65.05
	S	1	0.97	11	10.68	91	88.35
Cephems	KF	9	8.74	14	13.59	80	77.67
Fluoroquinolones	CIP	15	14.56	35	33.98	53	51.46
	ENR	8	7.77	22	21.36	73	70.87
Glycopeptides	TEC	63	61.17	23	22.33	17	16.50
	VA	29	28.16	36	34.95	38	36.89
Macrolides, Streptogramins, Lincosamides	E	8	7.77	44	42.72	51	49.51
	QD	11	10.68	22	21.36	70	67.96
	DA	8	7.77	4	3.88	91	88.35
Penicillins	OX	0	0.00	0	0.00	103	100.00
	AMC	80	77.67	12	11.65	11	10.68
	AMP	78	75.73	1	0.97	24	23.30
Tetracyclines	TE	27	26.21	20	19.42	56	54.37
	TCG	36	34.95	25	24.27	42	40.78

Legend. RD, rifampicin; C, chloramphenicol; LZD, linezolid; F, nitrofurantoin; W, trimethoprim; N, neomycin; CN, gentamicin; S, streptomycin; KF, cephalothin; CIP, ciprofloxacin; ENR, enrofloxacin; TEC, teicoplanin; VA, vancomycin; E, erythromycin; QD, quinupristin–dalfopristin; DA, clindamycin; OX, oxacillin; AMC, amoxicillin–clavulanic acid; AMP, ampicillin; TE, tetracycline; TIG, tigecycline.

**Table 2:** Antibiotic resistance of the analyzed *Enterococcus* isolates in relation to bird provenience and category and to bacterial species.

		Number of *Enterococcus* Isolates (%)							
		Examined	Vancomycin Resistant	Ampicillin MIC ≥64 mg/L	HLSR	HLGR	MDR	XDR	PDR
Total		103	5 (4.85)	6 (5.83)	34 (33.01)	15 (14.56)	79 (76.69)	20 (19.41)	3 (2.91)
Provenience of birds	Hunting activity	42	1 (2.38)	0 (0.00)	3 (7.14)	2 (4.76)	26 (61.90)	15 (35.71)	1 (2.38)
	Recovery center	61	4 (6.56)	6 (14.28)	31 (50.82)	13 (21.31)	53 (86.89)	5 (8.20)	2 (3.28)
Avian category	Synanthropic birds	18	0 (0.00)	2 (11.11)	9 (50)	2 (11.11)	14 (77.78)	2 11.11)	2 (11.11)
	Raptors	17	2 (11.76)	2 (11.76)	10 (58.82)	4 (23.53)	16 (94.12)	1 (5.88)	0 (0.00)
	Aquatic birds	68	3 (4.41)	2 (2.94)	15 (22.06)	9 (13.24)	49 (72.06)	17 (25.00)	1 (1.47)
Bacterial species	*E. faecium*	62	3 (4.84)	5 (8.06)	24 (38.71)	7 (11.29)	46 (74.19)	13 (30.97)	3 (4.84)
	*E. faecalis*	36	2 (5.56)	0 (0.00)	8 (22.22)	7 (19.44)	29 (80.56)	7 (19.44)	0 (0.00)
	*E. avium*	3	0 (0.00)	0 (0.00)	1 (33.33)	0 (0.00)	2 (66.67)	0 (0.00)	0 (0.00)
	*E. durans*	2	0 (0.00)	1 (50.00)	1 (50.00)	1 (50.00)	2 (100)	0	0 (0.00)

**Table 3:** Results of the molecular analyses in relation to the bacterial species, provenience, and category of birds.

		Provenience of Birds		Avian Category			Bacterial Species			
	Total	Hunting Activity	Recovery Center	Synanthropic Birds	Raptors	Aquatic Birds	*E. durans*	*E. avium*	*E. faecalis*	*E. faecium*
Examined	103	42	61	18	17	68	2	3	36	62
*vanA*	0 (0.00%)	0 (0.00%)	0 (0.00%)	0 (0.00%)	0 (0.00%)	0 (0.00%)	0 (0.00%)	0 (0.00%)	0 (0.00%)	0 (0.00%)
*vanB*	0 (0.00%)	0 (0.00%)	0 (0.00%)	0 (0.00%)	0 (0.00%)	0 (0.00%)	0 (0.00%)	0 (0.00%)	0 (0.00%)	0 (0.00%)
*aac(6′)-Ie-aph(2″)-Ia*	6 (5.82%)	0 (0.00%)	6 (9.84%)	3 (16.67%)	1 (5.88%)	2 (2.94%)	0 (0.00%)	0 (0.00%)	2 (5.56%)	4 (6.45%)
*ant(6)-Ia*	17 (16.50%)	2 (4.76%)	15 (24.59%)	5 (27.78%)	3 (17.65%)	9 (13.24%)	0 (0.00%)	1 (33.33%)	5 (13.89%)	11 (17.74%)
*aac(6′)-li*	55 (53.39%)	23 (54.76%)	32 (52.46%)	10 (55.56%)	11 (64.71%)	34 (50.00%)	0 (0.00%)	2 (66.67%)	3 (8.33%)	50 (80.65%)
*tet(M)*	34 (33.01%)	8 (19.05%)	26 (42.62%)	7 (38.89%)	7 (41.18%)	20 (29.41%)	0 (0.00%)	1 (33.33%)	11 (30.56%)	22 (35.48%)
*tet(L)*	9 (8.74%)	2 (4.76%)	7 (11.48%)	0 (0.00%)	3 (17.65%)	6 (8.82%)	0 (0.00%)	0 (0.00%)	4 (11.11%)	5 (8.06%)
*tet(O)*	2 (1.94%)	0 (0.00%)	2 (3.28%)	0 (0.00%)	0 (0.00%)	2 (2.94%)	0 (0.00%)	0 (0.00%)	2 (5.56%)	0 (0.00%)
*tet(K)*	0 (0.00%)	0 (0.00%)	0 (0.00%)	0 (0.00%)	0 (0.00%)	0 (0.00%)	0 (0.00%)	0 (0.00%)	0 (0.00%)	0 (0.00%)
*Int-Tn*	12 (11.65%)	4 (3.88%)	8 (7.77%)	3 (16.67%)	3 (17.65%)	6 (8.82%)	0 (0.00%)	1 (33.33%)	2 (5.56%)	9 (14.52%)

**Table 4:** Tested birds in relation to their provenience and species.

Provenience of Birds	Avian Category	Common Name	Scientific Name	Number of Tested Animals
Recovery center	Raptors	Little owl	*Athene noctua*	5
		Peregrine falcon	*Falco peregrinus*	4
		Common kestrel	*Falco tinnunculus*	3
		Eurasian buzzard	*Buteo buteo*	3
		Long-eared owl	*Asio otus*	1
		Barn owl	*Tyto alba*	1
	Synanthropic birds	Common wood pigeon	*Columba palumbus*	1
		Common starling	*Sturnus vulgaris*	1
		European robin	*Erithacus rubecula*	1
		Song thrush	*Turdus philomelos*	2
		Hooded crow	*Corvus cornix*	1
		European turtle dove	*Streptopelia turtur*	7
		Domestic pigeon	*Columba livia*	5
	Aquatic birds	Yellow-legged gull	*Larus michahellis*	26
Hunting activity	Aquatic birds	Eurasian teals	*Anas crecca*	29
		Mallard	*Anas platyrhynchos*	3
		Shoveler	*Spatula clypeata*	8
		Pintail	*Anas acuta*	1
		Mandarin duck	*Aix galericulata*	1

## Data Availability

The data presented in this study are available within the article.

## Supplementary Materials

The following supporting information can be downloaded at: https://www.mdpi.com/article/10.3390/antibiotics11070852/s1, Table S1: primers and protocols employed for molecular analyses. References [51,52,53,54,55] are cited in the Supplementary Materials.
