# Supplementary material for: Antimicrobial-Resistant Enterococcus spp. in Wild Avifauna from Central Italy
Source: Antibiotics (Basel). 2022 Jun 24;11(7):852. doi: 10.3390/antibiotics11070852 (PMC9311988; doi:10.3390/antibiotics11070852)
Supplement: Supplementary file 1 [file antibiotics-11-00852-s001.zip › antibiotics-1775927-supplementary.pdf]

antibiotics

MDPI

Supplementary material

Antimicrobial-Resistant *Enterococcus* spp. in Wild Avifauna from Central Italy

Giulia Cagnoli [1], Fabrizio Bertelloni [1,*], Paolo Interrante [1], Renato Ceccherelli [2], Margherita Marzoni [1] and Valentina Virginia Ebani [1,3]

Table S1. Primers and protocols employed in molecular analyses.

Resistance to		Target gene	Sequences (5′- 3′)	Annealing Temp. (°C)	Amplicon size (bp)	References
Vancomycin		*vanA*	F: GGGAAAACGACAATTGC<br>R: GTACAATGCGGCCGTTA	54	732	[1]
		vanB	F: ATGGGAAGCCGATAGTC<br>R: GATTTCGTTCCTCGACC	54	635	
Aminoglycoside	*HLGR*	*aac(6′) -Ie-aph(2′′)-Ia*	F: CAGAGCCTTGGGAAGATGAAG<br>R: CCTCGTGTAATTCATGTTCTGGC	56 (55)	348	[2]
	HLSR	*ant(6)-Ia*	F: CGGGAGAATGGGAGACTTTG<br>R: CTGTGGCTCCACAATCTGAT	55	563	[3]
	Gentamy-cin and other	*aac(6′)-Ii*	F: TGGCCGGAAGAATATGGAGA<br>R: GCATTTGGTAAGACACCTACG	55	410	
Tetracycline		*tet*(M)	F: GTGGACAAAGGTACAACGAG<br>R CGGTAAAGTTCGTCACACAC	61 (62)	406	[4]
		tet(L)	F: TGGTGGAATGATAGCCCATT<br>R: CAGGAATGACAGCACGCTAA	61 (62)	229	
		tet(O)	F: AACTTAGGCATTCTGGCTCAC<br>R: TCCCACTGTTCCATATCGTCA	61 (62)	515	

	tet(K)	F: GATCAATTGTAGCTTTAGGTGAAGG<br>R: TTTTGTTGATTTACCAGGTACCATT	61 (62)	155	
Int-Tn	(Tn*916*/Tn*1545*)	F: GCGTGATTGTATCTCACT<br>R: GACGCTCCTGTTGCTTCT	50	1028	[5]

References

1. Dutka-Malen, S.; Evers, S.; Courvalin, P. Detection of glycopeptide resistance genotypes and identification to the species level of clinically relevant enterococci by PCR. *J. Clin. Microbiol.* **1995**, *33*, 24.
2. Vakulenko, S.B.; Donabedian, S.M.; Voskresenskiy, A.M.; Zervos, M.J.; Lerner, S.A.; Chow, J.W. Multiplex PCR for Detection of Aminoglycoside Resistance Genes in Enterococci. *Antimicrob. Agents Chemother.* **2003**, *47*, 1423.
3. Kobayashi, N.; Mahbub Alam, M.; Nishimoto, Y.; Urasawa, S.; Uehara, N.; Watanabe, N. Distribution of aminoglycoside resistance genes in recent clinical isolates of Enterococcus faecalis, Enterococcus faecium and Enterococcus avium. *Epidemiol. Infect.* **2001**, *126*, 197.
4. Malhotra-Kumar, S.; Lammens, C.; Piessens, J.; Goossens, H. Multiplex PCR for Simultaneous Detection of Macrolide and Tetracycline Resistance Determinants in Streptococci. *Antimicrob. Agents Chemother.* **2005**, *49*, 4798.
5. Doherty, N.; Trzcinski, K.; Pickerill, P.; Zawadzki, P.; Dowson, C.G. Genetic Diversity of the tet(M) Gene in Tetracycline-Resistant Clonal Lineages of Streptococcus pneumoniae. *Antimicrob. Agents Chemother.* **2000**, *44*, 2979.
